# Washing or Bathing, Therapeutics of

**Published:** 1884-05

**Authors:** 


### WASHING OR BATHING.

AVERSION TO WASHING: Amm. c., *Amm. m.*, ANT. CRUD., Baryt., BELL., BORAX, Bov., BRY., CALC., CANTH., CARBO VEG., CHAM., CLEM., *Con.*, *Dulc.*, *Kali*, LAUR., Lyc., Magn. c., Merc., MEZER., Mur. ac., Natr. c., NITRUM, *Nitr. acid*, *Nux mos.*, Nux vom., Phosph., PULS., RHUS, SARSAP., SEPIA, Sil., SPIG., Stann., STAPH., STRONT., SULPH., *Sul. acid.*, *Zinc.*

AGGRAVATION FROM WASHING (in general): Æsc-hipp., Æthusa, AMM. C., Amm. m., ANT. CRUD., Baryt., BELL., Borax, Bov., *Bry.*, CALC., CANTH., CARBO VEG., *Caust.*, CHAM., CLEM., Con., Dulc., *Kali c.*, Kobalt., Laur., Magn. c., Mang., MERC.., Mezer., Mur. acid, Natr. c., NITRUM, NITR. ACID., Nux vom., *Phosph.*, Puls., RHUS, SARSAP., SEPIA, SPIG., Stann., Staph., STRONT., SULPH., *Sul. acid*, *Zinc.*

AMELIORATION FROM WASHING (in general): *Alum*, AMM. M., Ant. tart., *Apis.*, *Ars.*, ASARUM, Borax, Bry., Calc., Caust., Cham., CHELID., Euphr., *Fluor. acid.*, Helon., Laur., Magn. c., Mezer., Mur-acid, *Nux vom.*, PULS., *Rodod.*, *Sabad.*, Sepia, SPIG., Staph., Zinc.

BATHING aggravates: ANT-CRUD., *Calc.*, *Carbo veg.*, *Caust.*, Mang., NITR. ACID, RHUS, *Sepia*, Sulph.

— in cold water, aggravates: BELL., NITR. ACID, RHUS, *Sarsap.*, SEPIA.

— in sea water, aggravates: *Mag-mur.*, *Rhus*, Sepia, Zincum.

### SYMPTOMS IN FULL.

ÆSCULUS HIPP. Pricking, swollen feeling in hands after washing them.

ÆTHUSA. Sensation as if head, face, and hands were swollen; worse after washing; better coming into room.

ALUMINA. Acrid, profuse leucorrhœa, relieved by cold washing. Rhagades (on hands) worse in winter and from washing.

AMM. CARB. Nosebleed when washing face in morning. Hands look blue and veins distended after washing in cold water.

AMM. MUR. Eyes glued together in morning, with burning in canthi after washing.

ANT. CRUD. Child cries when washed in cold water, better when washed in warm water. Headache after bathing in a river, with weakness of limbs and aversion to food. Disposition to take cold about head after getting wet or bathing in cold water. *Diarrhœa from cold bath.*

APIS. Worse from getting wet through, but better from moistening or washing the affected part in cold water.

ARNICA. Nosebleed after washing face: (Amm. c., Antimon. sul. aur., Dros., Kali c., Kali b.)

ARSENIC. General aggravation from cold, except headache, which is temporarily relieved by cold washing, and permanently by walking in cold air.

BORAX. Washing chest with cold water affords relief from pains.

BROMIUM. Hæmorrhoids worse from cold or warm water, better from wetting with saliva.

BRYONIA. Facial neuralgia relieved by cold applications.

CALC. PHOS. Dullness with every headache; worse from mental exertions; better from cold washing.

CANTHARIS. Headache from washing or bathing. Warm applications relieve pains in knees.

CLEMATIS. Itching of skin worse from washing in cold water, from warmth of bed, and from wet poultices.

COLOCYNTH. Tensive, tearing pain, with heat and swelling, especially of left side of face; worse from touch or motion; better in perfect rest and from external warm applications.

CONIUM. Soreness as from excoriation in skin of face after washing and wiping face.

CYCLAMEN. Headache relieved by cold water applications.

EUPHRASIA. Rash on the face; itching in the warmth, becoming red and burning when moistened.

FERRUM. Neuralgia (facial) after washing and over-heating.

FLUOR. ACID. General heat, with nausea from slightest motion, with inclination to uncover, but mostly to wash in cold water.

HYDRASTIS. Eczema on margin of hair in front (of head); worse coming in from cold into warm room; oozes after washing.

KALI CARB. Nosebleed when washing the face; every morning at 9 A. M.

LAUROCERASUS. Rough, scaly skin between the fingers, with burning when touched by water.

LOBELIA INFLATA. Cold washing increases or causes pains; causes difficult breathing.

MAGN-MUR. Congestion of blood to the chest from bathing in the sea. Bloody expectoration brought on by sea bathing. Great weakness after sea bath.

MEPHITES. Feels less chilly in cold weather; feels pleasant after ice-cold washing.

MERC. IOD. RUB. Violent tearing in soles; feet swollen, sore to touch, worse around ankles; after washing the floor.

MEZEREUM. Better walking in open air, yet sensitive to cold air or to cold washing in the morning.

NATRUM CARB. Anxious in evening after a foot bath.

NATRUM MUR. Great inclination for open air and for washing in cold water.

NITRUM. Burning in the eyes, lachrymation and aversion to light especially in the morning; after washing in cold water.

NUX MOS. Abdominal colic, better from hot wet cloths; muscular rheumatism worse from cold wet cloths.

PHOSPHORUS. Toothache from washing clothes; from having hands in cold or warm water. Weight and throbbing in forehead on waking, better by cold washing, worse on stooping; sometimes lasts all day. Dullness in head, amel. by cold washing.

PHYTOLACCA. Tinea capitis; worse washing it when he is warm. Blotches on face; worse in P. M., after washing and eating.

PODOPHYLLUM. Diarrhœa (*i. e.*, stool) while being washed. Backache after washing, with prolapsus uteri.

PSORINUM. Pain as if brain had not room enough in forehead, when rising in morning; better after washing and eating.

PULSATILLA. Heat of right side, or on upper part of body; lessened by moving or washing.

SABADILLA. Mania; rage quieted only by washing the head in cold water.

SPIGELIA. Dull stitches from within outward, on top of head; worse from touch and after washing, but better while washing it.

SULPHUR. Toothache coming on in open air, or from least draught, or at night in bed, or from washing with cold water.
TABACUM. Cramps in single fingers, especially while washing; early morning.
TARENTULA. Stools three or four times daily, very dark, fetid, partly formed, containing much mucus, expelled with difficulty, and followed by smarting and burning at anus but no tenesmus; stools always occurred immediately on having the head washed.
THUJA. Face; skin hot and red, peels off when washed.
ZINCUM. Yellowish herpes in mouth from sea bathing.

